# Two types of nanoparticle-based bio-barcode amplification assays to detect HIV-1 p24 antigen

**DOI:** 10.1186/1743-422X-9-180

**Published:** 2012-08-31

**Authors:** Huahuang Dong, Jianli Liu, Hong Zhu, Chin-Yih Ou, Wenge Xing, Maofeng Qiu, Guiyun Zhang, Yao Xiao, Jun Yao, Pinliang Pan, Yan Jiang

**Affiliations:** 1 National HIV/HCV Reference Laboratory, National Center for AIDS/STD Control and Prevention, Chinese Center for Disease Control and Prevention, Beijing, The People’s Republic of China; 2 HIV Central Confirmatory Laboratory, Beijing Exit and Entry Inspection and Quarantine Bureau, Beijing, The People’s Republic of China; 3 Global AIDS Program-China office, US Centers for Disease Control and Prevention, Beijing, The People’s Republic of China

**Keywords:** Human immunodeficiency viruses, Bio-barcode amplification, p24 detection

## Abstract

**Background:**

HIV-1 p24 antigen is a major viral component of human immunodeficiency virus type 1 (HIV-1) which can be used to identify persons in the early stage of infection and transmission of HIV-1 from infected mothers to infants. The detection of p24 is usually accomplished by using an enzyme-linked immunosorbent assay (ELISA) with low detection sensitivity. Here we report the use of two bio-barcode amplification (BCA) assays combined with polymerase chain reaction (PCR) and gel electrophoresis to quantify HIV-1 p24 antigen.

**Method:**

A pair of anti-p24 monoclonal antibodies (mAbs) were used in BCA assays to capture HIV-1 p24 antigen in a sandwich format and allowed for the quantitative measurement of captured p24 using PCR and gel electrophoresis. The first 1 G12 mAb was coated on microplate wells or magnetic microparticles (MMPs) to capture free p24 antigens. Captured p24 in turn captured 1D4 mAb coated gold nanoparticle probes (GNPs) containing double-stranded DNA oligonucleotides. One strand of the oligonucleotides was covalently immobilized whereas the unbound complimentary bio-barcode DNA strand could be released upon heating. The released bio-barcode DNA was amplified by PCR, electrophoresed in agarose gel and quantified.

**Results:**

The in-house ELISA assay was found to quantify p24 antigen with a limit of detection (LOD) of 1,000 pg/ml and a linear range between 3,000 and 100,000 pg/ml. In contrast, the BCA-based microplate method yielded an LOD of 1 pg/ml and a linear detection range from 1 to 10,000 pg/ml. The BCA-based MMP method yielded an LOD of 0.1 pg/ml and a linear detection range from 0.1 to 1,000 pg/ml.

**Conclusions:**

When combined with PCR and simple gel electrophoresis, BCA-based microplate and MMPs assays can be used to quantify HIV-1 p24 antigen. These methods are 3–4 orders of magnitude more sensitive than our in-house ELISA-based assay and may provide a useful approach to detect p24 in patients newly infected with HIV.

## Introduction

The early stage of HIV infection is characterized by the absence of detectable antibodies and transient high viral load. Patients in this window period have a high probability of HIV transmission [1,2]. Virologic markers such as HIV-1 RNA, DNA and p24 antigen can be used to determine infection status. The direct detection of viral nucleic acids using PCR-based approaches is of high technical complexity and requires skilled personnel, equipment and laboratory infrastructure. It is also complicated by the high variation and continuing diversification of HIV viral genome and subtypes [3-5]. Detection of p24 antigen could serve as a viable alternative for the detection of infection during the window period [6-8] and in HIV-1 exposed infants [9,10].

ELISA has been previously used for HIV-1 p24 antigen detection. Due to the interference of antibodies in infected persons, conventional ELISA assays can detect p24 in approximately 10% asymptomatic infected persons and 30 to 40% patients with late stage AIDS-related syndrome [11]. Several modifications have been introduced to improve detection sensitivity by heat or acid disruption of antigen-antibody complexes [12], signal amplification based on tyramide [13] and immuno-PCR [14]. An ultra-sensitive BCA assay with GNPs was recently developed to detect proteins with special clinical significance [15]. To capture the antigen of interest, MMPs of 1 μm in diameter are coated with a mAb to the antigen. Addition of GNPs dually labeled with a second mAb and bio-barcode DNA oligomers results in the formation of an antigen, antibody, DNA oligomer complex. The amount of the DNA oligomers co-captured is directly proportional to the amount of the antigen and can be quantitatively measured by chip-based scanometric method [15-20] and PCR [21,22]. Here we describe the use of two types of BCA assay with simple PCR and gel analysis to achieve HIV-1 p24 quantification.

## Results

### Detection of HIV-1 p24 antigen by ELISA

**Figure 1:**
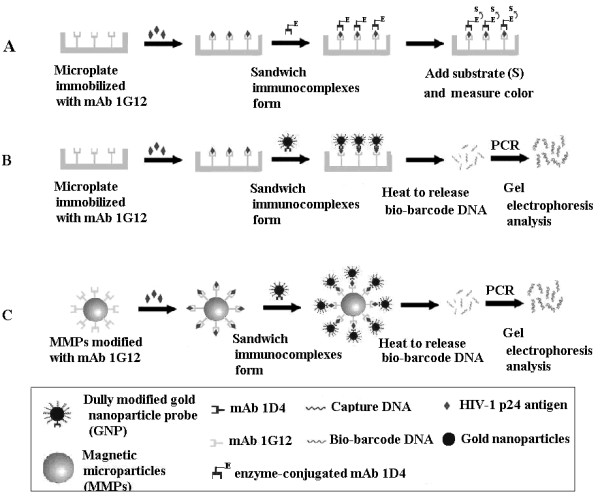
**Schematic illustrations of two biobarcode amplification (BCA) assays and ELISA assay to detect HIV-1 p24 antigen.** (**A**) ELISA, (**B**) Microplate [22] and (**C**) MMPs methods. The detailed description of the methods is described in Materials and Methods. The enzyme (E) used in the ELISA assay was horse radish peroxidase.

**Figure 2:**
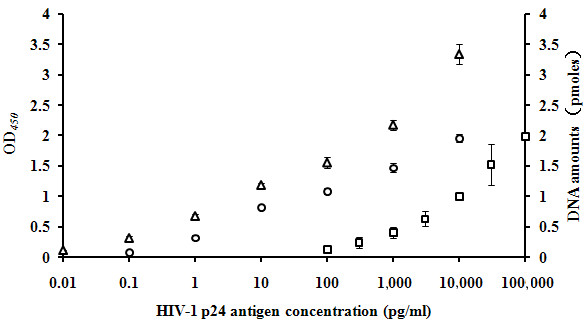
**Relative HIV-1 p24 antigen detection sensitivity using 2 BCA-based assays and an in-house ELISA assay.** The X axis represents serial dilutions (log_10_) of HIV-1 p24 antigen, the left Y axis is OD_*450*_ value for ELISA, and the right Y axis is the DNA amounts (pmoles) calculated from gel electrophoresis band of samples for the two BCA assays. Each data point represents the average of 3 independent determinations. Squares : ELISA, circles: microplate method, and triangles: MMPs method.

**Figure 3:**
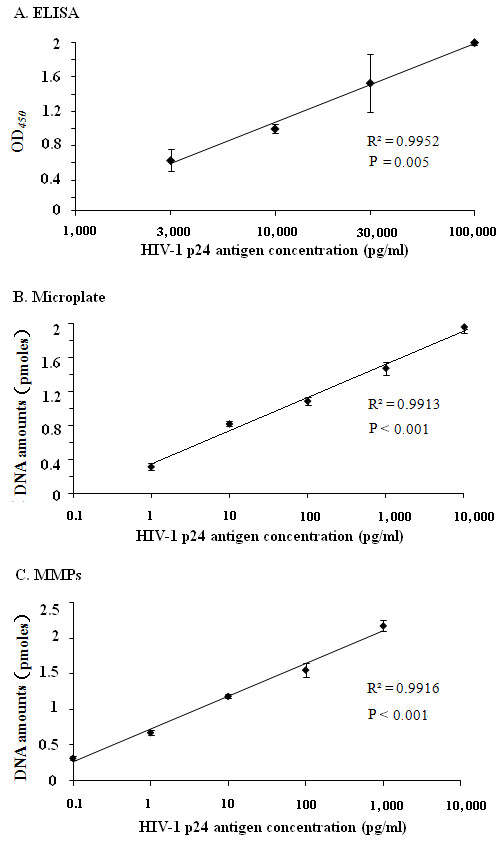
**Linear dynamic p24 detection ranges of three assays: (A) in-house ELISA, (B) BCA-based microplates and (C) BCA-based MMPs.** Each data point represents the average of three independent determinations. Statistical analysis of linear regression model between the signal value of each methods and the concentration of HIV-1 p24 are carried out with SPSS 16.0.

First, we examined the quantification of p24 antigen using an in-house ELISA assay with 1 G12 and 1D4 mAb pair (Figure 1A). Serial dilutions of p24 antigens from 100,000 to 100 pg/ml were added to the microplate wells precoated with 1 G12 mAb and then incubated with horseradish peroxidase labeled 1D4 mAb for colorimetric reaction. The average of 3 independent OD_450_ measurements of negative controls was 0.249 (standard deviation SD = 0.014) and the cutoff (CO) was 0.291 (= mean plus 3SD). The SD (Figure 2) and coefficient of variations (1.6–22.2%) of the data point range were small. The LOD was 1, 000 pg/ml (Figure 2). The signal and antigen response curve increased slowly and was linear in the range between 3,000 and 100,000 pg/ml (Figure 3A).

### PCR and gel detection of signal strand of bio-barcode DNA

**Figure 4:**
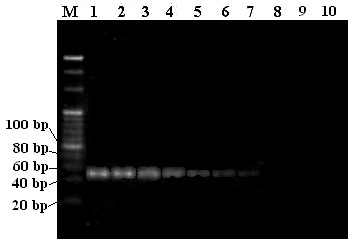
**Amplification detection of 10-fold diluted bio-barcode is revealed by 4% agarose gel electrophoresis.** The concentration of DNA in lane 1 was 0.005 uM and 5 μl of PCR DNA were used for gel electrophoresis. Lane M is the 20 bp ladder reference DNA marker (Takara Inc.). Lanes 1 to 9 represents amplicons 3 × 10^9^, 3 × 10^8^, 3 × 10^7^, 3 × 10^6^, 3 × 10^5^, 3 × 10^4^, 3 × 10^3^, 300, and 30 copies of signal bio-barcode DNA. Lane 10 represents negative control (PCR reaction mixture without input DNA).

To improve the p24 detection sensitivity, we employed two types of BCA methods to capture p24 and then used PCR and gel electrophoresis to measure the amount of amplified bio-barcode DNA (Figure 1 B and C). To demonstrate the utility of PCR and gel electrophoresis to measure the bio-barcode DNA oligomer, we used unbound free single-stranded bio-barcode DNA oligomer (3 × 10^9^ to 30 copies), amplified with a bio-barcode DNA specific primer pair for 25 cycles and examined the amplified product in a 4% agarose gel. As shown in Figure 4, clear DNA bands with anticipated 47 bp in length were detected in samples with input DNA of 3 × 10^9^ to 3,000 copies (lanes 1 ~ 7) but not in samples with input DNA of 300 copies and 30 copies (lanes 8 and 9, respectively). No bands or primer dimers were found after 25 cycles of PCR for the negative control (lane 10).

### Detection of HIV-1 p24 antigen by BCA using microplate

With the establishment of the method to measure bio-barcode oligomer using PCR and gel electrophoresis, we proceeded with the BCA-based microplate assay (Figure 1B). Serial dilutions (0.1 to 10,000 pg/ml) of HIV-1 p24 antigen were added onto microplate wells precoated with 1 G12 mAb followed by the addition of GNPs. The amount of bio-barcode DNA on the captured GNPs was measured by PCR and gel electrophoresis. The signal of the 47 bp PCR products in 3 independent experiments were measured. The cutoff value was 0.098 (mean plus 3 SD). The SD (Figure 2) and coefficient of variations (3.4–13.1%) of the entire data point range were small. The LOD of this assay was 1 pg/ml (Figure 2) and the linear detection range of p24 was from 1 to 10,000 pg/ml (Figure 3B).

### Detection of HIV-1 p24 antigen by BCA using MMPs

We further substituted the microplate with magnetic microparticles to increase the binding kinetics between the p24 antigen and capture mAb (Figure 1C). The measurement of p24 by PCR and gel electrophoresis remained the same as described above. The cutoff value was 0.125 (mean of 3 determinations and 3 SD). The SD (Figure 2) and coefficient of variations (2.5-10.3%) of the entire data point range were small. The LOD of this assay was improved to 0.1 pg/ml (Figure 2) and the linear detection range of p24 was from 0.1 to 1,000 pg/ml of p24 (Figure 3C).

## Discussion

BCA-based assays have become a new platform frequently used to detect trace proteins requiring high sensitivity detection [18,23,24]. It has been applied to the early diagnosis of cancer markers and infectious diseases such as prostate-specific antigen (PSA) [15,16], human chorionic gonadotropin and α-fetoprotein [25], amyloid-ß-derived diffusible ligand [26], interleukin-2 [23], Hantaan viral nucleocapsid protein [22] and HIV-1 p24 antigen [17,19,21]. Likewise, here we showed that BCA-based microplate and MMP assays coupled with PCR signal amplification offer greater detection sensitivity over an ELISA assay by 1,000 to 10,000 fold, respectively. The increase of signal is achieved by (1) the large number of bio-barcode DNA oligomer coated onto the GNPs [20,25] and (2) PCR amplification of the bio-barcode DNA released from the GNPs after specific p24 and mAb capture.

Several forms of signal amplification methods have been used including chip-based scanometric detection [15,19,25,26], colormetric method [23], and fluorescent approach [20]. Kim and colleagues used MMPs and compared real-time PCR and chip-based methods. They found that the two methods performed similarly and achieved a sensitivity of 0.1 pg/ml and a detection range from 0.1 pg/ml to 10,000 pg/ml [21]. Tang used a BCA-based microplate assay combined with chip-based method to achieve an LOD of HIV-1 p24 at 0.1 pg/ml with the linear range of 0.1–500 pg/ml [19]. In an attempt to simplify assay procedures and shorten the reaction time, Tang et al., used europium nanoparticles to replace GNPs, but the LOD increased from 0.1 to 0.5 pg/ml [17]. In this report, we showed that our MMP assay was reproducible with small SD and CV. It achieved 10-fold detection sensitivity over that of the microplate method. Our MMP assay has comparatively simpler operation and shorter reaction time than those reported earlier [21] and we achieved the same LOD and a wider linear range of 0.1 to 1,000 pg/ml in two hours.

One of the disadvantages of PCR-based methods is the potential of cross-contamination. Although this phenomenon was not observed in our study, measures to avoid PCR contamination should be strictly observed [27,28]. We had attempted the use of real-time PCR with SYBR Green to replace gel-based quantification but we found the magnitude of signal amplification over the intended detection range of p24 was suboptimal (data not shown). One of the important applications of p24 detection in China is to identify HIV-infected infants born to HIV-infected mothers. Currently there is no government-approved domestic HIV-1 p24 or nucleic acid-based technologies available. We will continue improving the current procedures by identifying a better mAb pair for the p24 and GNP complex formation and assembling a contamination free real-time PCR system for the accurate and rapid identification of HIV infection.

## Conclusions

Our current study demonstrated that when combined with PCR and gel analysis, BCA-based assays using microplates and MMPs can detect HIV-1 p24 antigen 3 to 4 orders of magnitude more sensitive than the conventional ELISA assay and offers a wide linear dynamic range. This assay may be useful in China to detect HIV infection in infants born to HIV-infected mothers.

## Materials and methods

### HIV p24 antigen and its monoclonal antibodies

HIV-1 p24 recombinant antigen was purchased from GenWay Biotech (San Diego, CA). Mouse anti-p24 monoclonal antibody, 1 G12 and 1D4, and their corresponding horseradish peroxidase-labeled mAbs were purchased from Jing Tiancheng biology company (Beijing, China). These two mAbs recognize different p24 epitopes. The 1 G12 mAb was used to capture p24 and 1D4 mAb was used as the secondary antibody for signal detection.

### Oligonucleotides and reagents

Bio-barcode DNA was 47-base in length and its sequence, 5’-CAGCTGGTCAGCAGAATGGTGTGACCCTCATGGCCGTCTTATCGGGT-3’, was derived from AT4G27630.1 (accessed December 25, 2011 at http://www.arabidopsis.org/index.jsp). Capture DNA sequence is complementary to the Bio-barcode DNA with an addition of an SH group and 15 A residues at its 5’ end. The forward primer (5’-CAGCTGGTCAGCAGAATGGTG-3’) and reverse primer (5’-ACCCGATAAGACGGCCATGAG-3’) were used for the PCR amplification of bio-barcode DNA. All oligonucleotides were purchased from Sangon Co. Ltd. (Shanghai, China). PCR reagents were purchased from TaKaRa Biotechnology Co., Ltd (Dalian, China). Gold nanoparticles of 30 nm in diameter and tosyl-activated magnetic microparticles (MyOne™ Dynabeads®) were purchased from Ted Pella Inc (Redding, CA) and Invitrogen (Carlsbad, CA), respectively.

### Preparation of functionalized MMPs

MyOne™ Dynabeads® were conjugated with 1 G12 mAb according to manufacturer’s protocol. Briefly, 40 μl 1 G12 mAb (1 mg/ml), 10 μl magnetic microparticles, 42 μl 3 M (NH_4_) _2_SO_4_ and 66 μl borate buffer (0.1 M, pH 9.5) were combined in a 1.5 ml tube and incubated at 37°C at 1400 oscillations per minute for 24 h. The MMPs were separated from unbound components magnetically. Three hundred μl of blocking buffer consisting of phosphate-buffered saline (PBS), pH 7.4, 10% BSA and 0.05% Tween 20 was added and the MMPs mixture was incubated at 37°C at 1400 oscillations per minute for another 24 h. At the completion of incubation, the MMPs were separated magnetically and were washed twice with 1 ml of magnetic-probe solution containing PBS pH 7.4, 0.1% BSA and 0.05% Tween 20, and resuspended in 200 μl of the same solution and stored at 4°C until use. Functionalized MMPs retained their antigen binding activity for 4–6 months

### Preparation of functionalized GNPs

GNPs functionalized with 1D4 mAb and the capture DNA oligomers were prepared as described previously [22]. Briefly, the pH of GNPs in aqueous solution (330 pM) was first adjusted to pH 9.2 with 1 N NaOH. One ml of the solution was incubated with 6 μg 1D4 mAb at 22°C with gentle shaking for 30 min, followed by the addition of 57.7 μg capture DNA oligomer at 10°C for 16 h. The salt concentration was then adjusted to 0.1 M using 2 M NaCl followed with the addition of 0.3 ml of 10% BSA. The mixture was incubated at 22°C for 30 min and then centrifuged at 10,000 rpm at 4°C for 30 min. The supernatant was removed and the GNPs were resuspended and washed once with 0.01 M PBS. The particles were resuspended with 400 μl PBS containing 65.5 μg of the signal DNA oligomers and allowed to hybridize at 37°C for 1 h. Finally, the GNPs were centrifuged at 10,000 rpm (Eppendorf centrifuge 5415 R, Germany) at 4°C for 30 min and then resuspended in PBS containing 0.01% (v/v) Tween 20 and 0.1% (w/v) BSA. The functionalized GNPs solution was stored at 4°C until use.

### Detection of HIV-1 p24 antigen by ELISA

Microplate strips (Corning Costar Inc., Lowell, MA) were coated with 100 μl per well of 1 G12 mAb (5 μg/ml in 0.5 M carbonate buffer) at 4°C for 24 h. After incubation, the strips were washed twice with PBS and then treated with 10% BSA at 37°C for 24 h to block the surface. The strips were washed again and then coated with 100 μl per well of serially diluted p24 antigen (ranged from 0.1 to 100,000 pg/ml) by incubating at 37°C for 1.5 h. Unbound antigens were washed 3 times with PBS. One hundred μl of 1,000 fold diluted HRP-labeled 1D4 was added to each well and the plate was incubated at 37°C for 1 h followed with 6 washes with PBS. One hundred μl of tetramethylbenzidine was then added to each well and the plate was incubated at 22°C for 15 min. The enzymatic reaction was terminated with the addition of 50 μl of 2 M H_2_SO_4_, and the color was quantified in an ELx808 plate reader (BioTek, Seattle, WA) at the wavelength of 450 nm.

### Detection of HIV-1 p24 antigen by BCA based on microplate

The assay scheme is shown in Figure 1B. The coating of BCA and blocking processes of microplates were the same as in ELISA. One hundred μl of 10 fold serially diluted HIV-1 p24 (0.1 to 10,000 pg/ml) in PBS containing 3% BSA was added into 1 G12 mAb coated wells. After incubation at 37°C for 60 min, the wells were washed 5 times with PBS containing 5 mM EDTA and 0.05% (v/v) and Tween 20 (PBSET). Then 100 μl GNPs (with a final concentration of 50 pM in 10% BSA) were added into each well and incubated at 37°C for 20 min. The wells were washed 7 times with PBSET and 3 times with PBS and once with 50 μl of Milli-Q distilled water. Finally, the wells were membrane sealed and heated in a water bath at 80°C for 5 min to release the captured signal DNA oligomers.

PCR amplification was performed by adding 1 μl of the free bio-barcode DNA solutions as templates. Total volume of the PCR reaction mixture was 25 μl containing 2.5 μL 10x Ex Taq Buffer (Mg^2+^), 2 μl dNTPs (2.5 mM each), 0.5 μl each of forward and reverse primers (with final concentration of 20 μM each), 1 μl Bio-barcode DNA, and 0.8 unit of TaKaRa Ex Taq polymerase. PCR reaction was run on Veriti 96-well thermal Cycler (Applied Biosystems, Foster City, CA) with the following temperature profile: initial denaturation, 95°C, 1 min; 25 cycles of denaturation (95°C, 30 s), annealing (54°C, 30 s), and extension (72°C, 45 s); and final extension (72°C, 5 min). Five μl of the PCR product was electrophoresed in a 4% agarose gel and the signal of the amplified 47-bp bio-barcode DNA was measured using GeneGenius Gel Imaging System (Syngene, Cambridge, UK) and BandScan version 5.0 [29,30]. The 100 bp DNA band of the 20 bp DNA Ladder (Takara, Dalian, China) standard with known input amount prior to gel electrophoresis was used as the DNA reference. The gray scale of electrophoresed 47 bp product was directly compared with the reference and its DNA amounts (in pmoles) were automatically determined by BandScan.

### Detection of HIV-1 p24 antigen by BCA based on MMPs

The assay scheme is shown in Figure 1C. One hundred μl of ten-fold serial dilutions of HIV-1 p24 antigen (10,000 ~ 0.01 pg/ml) in 3% BSA, 0.2% Tween 20, PBS (pH 7.4) and 40 μl diluted functionalized MMPs (1:100 diluted in PBS containing 1% BSA and 0.2% Tween 20) were mixed in a tube of 1.5 ml for 60 min (1,400 oscillations/min) at 37°C, followed by 5 washes with PBS containing 0.1% BSA, 0.05% Tween 20. After magnetic separation, 0.5 μl functionalized GNPs and 3.3 μg tRNA (66 mg/ml, Sigma-Aldrich, Inc., St. Louis, MO) in PBS containing 10% BSA and 0.05% Tween 20 in a total volume of 200 μl were added in this reaction, followed by mixing for 20 min (1400 oscillations/min). The mixture was washed 8 times with PBS containing 0.1% BSA and 0.05% Tween 20 and 4 times with PBS. After magnetic separation, 50 μl of Milli-Q distilled water was added and the tubes were incubated at 80°C for 5 min to release bio-barcode DNA. One μl of the DNA solution was used for PCR amplification. PCR products were quantified as described in the previous section.

## Competing interests

The authors declare no competing interests.

## Authors’ contributions

HD developed the design of the study, performed the experiments, and drafted the manuscript. CO contributed scientific input and manuscript preparation. JL participated in the design of the study and part of the experiments. HZ, GZ, GX, YX, FQ, JY, and LP participated in acquisition, analysis, and interpretation of data. YJ supervised the studies. All authors read and approved the final manuscript.
